# Affordability of comprehensive community health worker programmes in rural sub-Saharan Africa

**DOI:** 10.1136/bmjgh-2017-000391

**Published:** 2017-09-25

**Authors:** Celia Taylor, Frances Griffiths, Richard Lilford

**Affiliations:** Division of Health Sciences, Warwick Medical School, University of Warwick, Coventry, UK

**Keywords:** affordability, community health workers, sub-Saharan Africa

## Abstract

**Introduction:**

Community health worker (CHW) programmes have low costs per person served and are central to achieving universal healthcare. However, their total cost is high and the target of one million CHWs for sub-Saharan Africa by 2015 was not met. We consider the affordability of rural CHW programmes by estimating total programme costs relative to national healthcare expenditure at different CHW salaries and resources available for healthcare.

**Methods:**

We combine an existing source of rural CHW programme costs with World Bank data to estimate relative CHW programme costs in 37 countries. We consider three ‘salaries’ (CHWs as volunteers, paid the local equivalent of US$80 per month and paid the national minimum wage) and four potential healthcare budgets (both actual and Abuja declaration allocations alone and increased by external funding received and potential foreign aid, respectively). Costs are shown in 2012 nominal US$.

**Results:**

With CHWs paid the local equivalent of US$80 per month and financed from existing central government healthcare budgets, the median relative cost of a CHW programme would be 27% of the healthcare budget. While less than 2.5% in five countries (Botswana, Equatorial Guinea, Gabon, Namibia and South Africa), this relative cost would exceed 100% in three (Chad, Eritrea and Niger). There is a strong negative linear relationship (R^2^=0.83, p<0.001) between the natural logs of gross domestic product (GDP) per capita and affordability. In 23 countries with GDP per capita under US$1200, the cost of a CHW programme would exceed 12% of actual healthcare spending if CHWs were paid US$80 per month.

**Conclusion:**

CHWs may be a stepping stone to universal access to professional healthcare, but there is high variability in the affordability of CHW programmes across sub-Saharan Africa. In many countries, such programmes are not yet affordable unless significant foreign aid is received.

**Key questions**

**What is already known about this topic?**

- Many countries in sub-Saharan Africa are struggling to meet the WHO targets for skilled healthcare personnel.
- Community health workers (CHWs) can provide basic healthcare, helping to relieve the shortage of skilled healthcare personnel.
- There is currently a shortage of CHWs across sub-Saharan Africa.

**What are the new findings?**

- If CHWs are paid the local equivalent of US$80 per month, a comprehensive rural programme would cost a median of 27% of 2012 central government healthcare spending.
- The high total costs of CHW programmes—even with volunteer CHWs—mean that they are unlikely to be affordable for some countries without significant external aid for healthcare.
- Affordability is most likely in countries with a per-capita gross domestic product (GDP) exceeding US$5000 and least likely if GDP per capita is less than <US$1200.

**Recommendations for policy**

- CHW programme ambitions may need to be scaled back in some countries.
- Reassurance of sustained support needs to be sought from external donors, lenders and internal decision-makers.
- Small-scale CHW programmes may be of benefit to test out optimal approaches to programme design in different settings.

## Introduction

Community health workers (CHWs) provide basic health promotion and healthcare within the communities in which they live. They are supported by the health system but are not necessarily a formal part of it.^1^ CHWs are often cited as part of the solution to the shortage of health workers and lack of universal access to healthcare in low-income settings^2 3^ and feature prominently in the WHO’s Workforce 2030 strategy for Human Resources for Health.^4^ They have two distinguishing features when compared with other potential providers of healthcare: being from the community in which they serve engenders trust from their clients, particularly when the community have been involved with their selection^5^ and, by travelling to clients’ homes, they reduce the need for long, difficult and/or expensive journeys to healthcare facilities. There is accumulating evidence that CHWs can provide individual health interventions effectively, for example, screening for and treatment of tuberculosis and promoting immunisation uptake.^6 7^ Many CHW programme providers are now starting to increase the scope of CHWs’ activities, for example, with CHWs providing integrated community case management, which includes screening for malnutrition and detection and treatment of malaria, pneumonia and diarrhoea in children.^8 9^

There is some evidence that these more formal, integrated CHW programmes can be a cost-effective component of health services, particularly for child health outcomes,^10 11^ and modelling shows the potential high long-run economic returns from investing in CHW programmes.^8^ However, by September 2016, the campaign to have one million CHWs in rural sub-Saharan Africa by the end of 2015 had counted just 332 000 (data available for 37 countries).^12^ There are three potential economic reasons for the shortfall. First, the total cost of initial scale-up and maintenance of CHW programmes is high: McCord and colleagues estimate annual maintenance costs of US$3.4 billion in 2012 prices per year across sub-Saharan Africa.^13^ Second, CHW programmes have to compete with other priorities for limited government resources, such as other cadres of health personnel. Third, even where financial resources are available, there are inevitable delays before CHWs can start working including the time taken to design appropriate programmes, build an effective supply chain and recruit and train CHWs.

One particularly malleable component of CHW programme costs is the incentive or reward paid to CHWs for their time and effort. Rewards vary widely across programmes. Of the CHWs enumerated in the one million CHWs campaign, 13% were reported as being salaried, 67% received a combination of monetary and non-monetary incentives, 18% received non-monetary incentives and 2% received no incentives at all.^12^ There can also be different ‘cadres’ of CHW within a health system: in Ethiopia, for example, salaried health extension workers are supported by a volunteer health development army. Although paying CHWs has generally become the accepted standard,^4 8 14^ it has also been argued that ‘financial incentives that are too low, irregularly paid or discontinued due to a lack of sustainable programme financing may result in more of a disincentive to CHWs than no payment at all’.^15^ A properly set-up CHW programme staffed by volunteer CHWs could therefore be appropriate where the alternative is no CHW programme at all given the local health and economic returns that could be realised.

The resources available to fund CHW programmes depend on total government expenditure, the proportion of such expenditure allocated to healthcare in general (and a CHW programme in particular) and the availability of external funding, including from donors, low-cost loans and human capital bonds repaid over an extended period based on future contingent savings.^8^ However, external sources of finance may not be reliable and may divert national resources away from healthcare, rather than supplementing it.^16–19^ There is also the possibility that aid is misused and does not reach its intended destination. The long-term optimal position would therefore be that nations can fund their own CHW programmes without requiring external aid.

The cost of a CHW programme and the resources available to pay for it therefore influence, at least in part, the scale of the programme in any particular country. The purpose of this paper is to analyse how the affordability of comprehensive CHW programmes would be affected by varying CHWs’ salaries and the resources available to pay for them. We also examine the relationship between the level of economic development in a country (proxied by gross domestic product (GDP) per capita) and the affordability of a CHW programme. Our work complements that of Bossert and Ono,^17^ who challenge the affordability of the WHO target of 2.3 physicians, nurses or midwives per 1000 population, and extends that of Oxford Policy Management,^20^ which estimated that a CHW programme in Pakistan providing 74% population coverage would require 27% of total government healthcare spending to be devoted to CHWs. As in this study in Pakistan, we provide a *relative* analysis to show CHW programme costs as a proportion of the public funding available for healthcare as a whole. By doing so across all mainland countries of sub-Saharan Africa, we are able to illustrate how the challenge of financing a CHW programme varies across countries. This multi-national approach enables us to explore the extent to which this variation can be explained by the level of economic development in each country.

## Methods

### Scope

We aimed to consider the 43 countries in mainland sub-Saharan Africa included in McCord *et al*’s detailed CHW programme costing study cited above.^13^ Because we draw on McCord *et al*’s study for CHW programme costs, our work similarly focuses on CHW programmes for rural populations. As no data on actual or potential healthcare budgets were available for Angola, Central African Republic, Djibouti, Guinea-Bassau, Somalia or Zambia, these six countries were excluded, leaving 37 for analysis. We take the position that CHW programmes are organised and financed by national governments. This implies that our work is at country level, although we recognise that there may be differences in CHW programmes within countries. All values are shown in nominal US$ at 2012 prices.

### Defining a full-scale comprehensive CHW programme for the rural population

By ‘full scale’, we mean that the CHW programme in each country provides universal access for all of the rural population at a ratio of 1 CHW to 650 rural community members, as in the costing study of McCord *et al*.^13^ By ‘comprehensive’, we mean that each CHW is trained and subsequently undertakes the following activities (with regular supervision): provision of oral rehydration salts and zinc for diarrhoea, amoxicillin for pneumonia and artemisinin-based combination therapy for malaria following diagnosis by the CHW; monitoring for undernutrition; and screening for tuberculosis.^13^

### CHW salary costs

We consider three potential ‘salaries’ for CHWs, with each representing the total monetary value of CHWs’ remuneration package, which may include incentives such as loans or free mobile phones. These are:

1. CHWs as volunteers (no salary).
2. CHWs paid an average of US$80 per month across countries as in McCord *et al*,^13^ regardless of hours actually worked. The actual value in each country reflects the relative price level in that country, determined using the World Bank’s price level ratio of purchasing power parity conversion factor (for GDP) to market exchange rate for 2012.^21^
3. CHWs paid the national minimum wage (generally based on a 40-hour week^22^) in each country, based on International Labour Organization’s compendium,^23^ which includes wage rates for the manufacturing sector or for unskilled workers, in the capital or major city (ideally we would use data for those in rural areas that are likely to be lower). Data are available for 30 countries. Values are converted from local currency units to US$ using World Bank exchange rates.^21^

These three salary levels closely approximate the three groups of CHWs identified in Olaniran *et al*’s systematic review (volunteer lay workers, level 1 paraprofessionals who are given an allowance or stipend and level 2 paraprofessionals who are salaried).^24^

### Non-CHW salary programme costs

We use the non-CHW salary costs of sustaining a full-scale, comprehensive CHW programme provided by McCord and colleagues.^13^ The costs in the paper are shown in nominal US$, and we assume that they reflect 2012 values. The mean non-CHW salary cost per full-time CHW estimated in this study was US$2190 per year. This figure includes CHW supervisor salaries, CHW training, equipment, medical supplies and ‘CHW-enablers’ (eg, bicycle, mobile phone, backpack and consumables), but not the knock-on costs or savings that may arise in other parts of the health service. In the costing by McCord *et al*, supervisor salaries and programme overheads were proportional to CHW salary. We consider them fixed within each country (ie, supervisors are paid US$X and overheads are US$Y regardless of CHWs’ salaries, with X and Y dependent on local price levels) because our focus is on the effect of different CHW salaries on affordability.

### Total healthcare budget

We use four potential total national healthcare budgets for each country:

1. Actual national budget: actual central government (public) spending on healthcare in 2012.^21^ This may include some overseas aid for capital projects (personal correspondence with World Bank).
2. Target national budget: 15% of central government spending in 2012,^21^ the target for healthcare spending in the 2001 Abuja Declaration.^25^
3. Actual national budget plus external resources for health (funds or services that are provided by entities not part of the country in question).^21^
4. Target national budget plus 2% of 2012 GDP,^21^ which reflects the maximum potential external funding for healthcare (2% was the maximum reported to have obtained across all countries in Bossert and Ono’s study^17^).

### Assessment of affordability

We calculated the percentage of each of the four total healthcare budgets that would need to be spent on a full-scale comprehensive CHW programme for the rural population at each of the three CHW ‘salaries’, giving 12 scenarios in all. The cost of a CHW programme is therefore considered *relative* to total current/possible healthcare spending (excluding any contributions from households or private organisations). The lower the percentage of the healthcare budget required to fund it, the more affordable the CHW programme.

We have not set an explicit CHW programme spending threshold because no government in sub-Saharan Africa currently finances a full-scale comprehensive CHW programme entirely from public funds, and hence, there is no guide as to what this threshold should be. This implies that any CHW programme in sub-Saharan Africa, if fully funded by a national government, would require an increase in the resources available for healthcare, a shift in the current budget allocation away from another part of the health service and/or efficiency gains in those other parts.

### Explaining variability in affordability using differences in levels of economic development

To examine the relationship between the level of economic development and CHW programme affordability, we plot the relative cost of a programme paying CHWs a salary of US$80 per month funded by actual national budgets (budget 1) against 2012 GDP per capita.^21^

## Results

### CHW salaries

The median monthly minimum wage across the 30 countries with data available was US$63, with a range from US$2 in Uganda to US$294 in Gabon. The local equivalent of US$80 is higher than the minimum wage in 19 of these 30 countries and is therefore the highest cost scenario for most countries. However, the overall mean difference in these two wage rates is small because minimum wages are negatively skewed. In the US$80 scenario, salary costs are a median of 30% of total programme costs (compared with 25% with minimum wages and, of course, 0% when CHWs are volunteers).

### Healthcare budgets

Only four countries spent more than the Abuja Declaration target of 15% of central government expenditure on healthcare in 2012 (Ethiopia, Malawi, Swaziland and Tanzania); all others fell short of this target, implying that budget 2 exceeds budget 1. Ten countries actually received more than 2% of GDP as aid for healthcare. In general, however, the lowest budget for healthcare analysed in this paper is actual central government spending on healthcare without any external funding/aid (budget 1), while the highest is 15% of central government expenditure plus 2% of GDP (budget 4).

### Relative affordability of a full-scale, comprehensive CHW programme for the rural population

Table 1 summarises our assessment of CHW programme affordability for each of the 12 scenarios. In general, affordability is lowest when the programme is funded from central government expenditure on healthcare without any aid (budget 1). When CHWs are paid the local equivalent of US$80 per month from this budget, the median (IQR) relative cost of a CHW programme was 27.3% (13.6%–58.8%) of the total healthcare budget and exceeds 100% in three countries (Chad, Eritrea and Niger). In contrast, CHW programmes are much more likely to be affordable if CHWs are volunteers and the programme is funded from the Abuja declaration target spending plus 2% of GDP (budget 4). In this scenario, the median (IQR) of the relative cost was 6.7% (2.4%–12.6%) of the total healthcare budget.

**Table 1:** Affordability of CHW programmes for each salary and healthcare budget scenario (shown as the median (IQR) [range] percentage of healthcare budget required to fund an ongoing CHW programme across countries)

	CHW salary		
Budget available for healthcare	US$80/month equivalent (37 countries)	Minimum wage (30 countries)	Volunteers (37 countries)
Budget 1: actual budget	27.3 (13.6 to 58.8) [0.6 to 126.2]	27.7 (14.9 to 43.4) [1.1 to 138.8]	19.5 (9.3 to 42.3) [0.4 to 90.9]
Budget 2: target budget: Abuja declaration 15% of central government expenditure	18.4 (7.9 to 38.1) [0.3 to 74.1]	20.1 (9.1 to 30.7) [0.6 to 61.4]	13.1 (5.8 to 58.0) [0.2 to 54.0]
Budget 3: actual budget+external resources for health	14.9 (8.8 to 25.8) [0.6 to 73.3]	14.2 (8.5 to 20.4) [1.1 to 71.5]	10.3 (6.5 to 19.3) [0.4 to 52.1]
Budget 4: target budget+2% GDP	9.4 (3.8 to 18.1) [0.2 to 32.1]	11.5 (4.2 to 17.1) [0.3 to 29.5]	6.7 (2.4 to 12.6) [0.1 to 24.2]

CHW, community health worker; GDP, gross domestic product.

### Variation in affordability as a function of per-capita GDP

**Figure 1:**
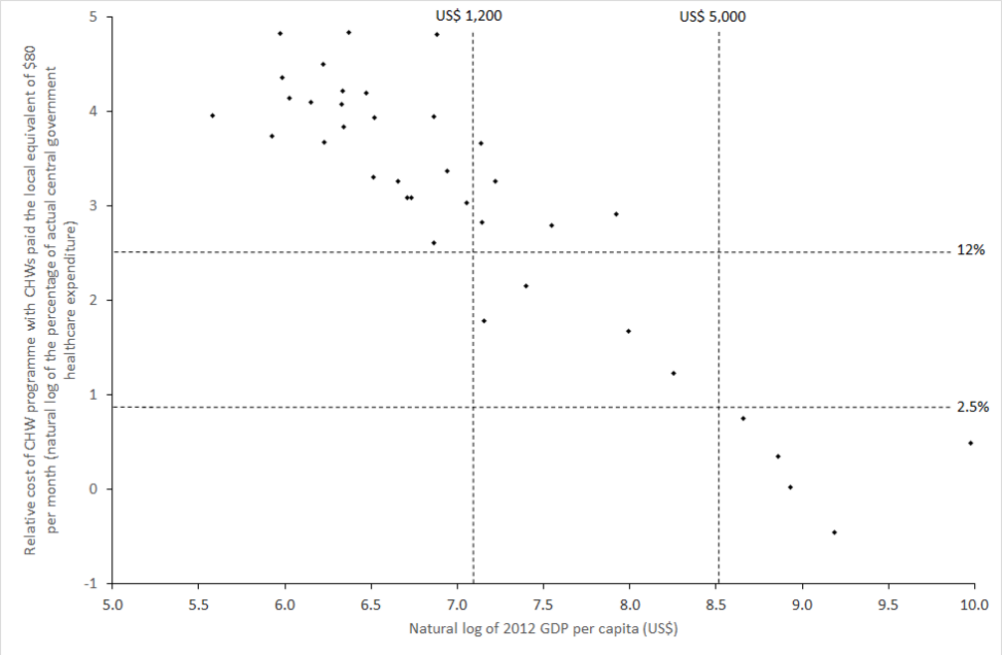
Relationship between GDP per capita and CHW programme affordability (percentage of actual central government spending on healthcare required to fund a CHW programme with CHWs paid US$80 per month), both on a log scale. CHW, community health worker; GDP, gross domestic product.

Figure 1 shows the relative cost of a CHW programme financed from actual central government healthcare spending (budget 1) with CHWs paid the equivalent of US$80 per month in relation to GDP per capita. Values of both variables are shown on a log scale given their large range and positive skew. There is a strong negative linear relationship (R^2^=0.83, p<0.001) between the natural logs of GDP per capita and affordability. The five countries with a GDP in excess of around US$5000/8.5 on the log scale (Botswana, Equatorial Guinea, Gabon, Namibia and South Africa) should be able to afford a comprehensive CHW programme without any external aid, with relative programme costs of up to around 2.5% of healthcare spending (0.9 on the log scale). Equatorial Guinea is an outlier on the chart (to the bottom right); among countries with a similar GDP per capita, it spends a relatively low amount on healthcare, which increases the relative cost of a CHW programme. There is no clear maximum GDP per capita at which a CHW programme is unaffordable, although relative programme costs are at least 12% of actual healthcare spending (2.5 on the log scale) in all 23 countries with a GDP per capita of less than US$1200/7.1 on the log scale.

## Discussion

Even the original WHO goal for the ratio of 2.3 skilled physicians, nurses or midwives per 1000 population is unrealistic for many countries,^17^ and the latest target has increased this ratio to 4.45.^26^ Expanded CHW programmes appear to offer the least expensive and a relatively quick route to the goal of universal access to healthcare in low-income and middle-income countries. However, our results suggest that CHW programmes could only be afforded by a small number of countries without significant increases in central government healthcare spending or the availability of external funding. As might be expected, the countries that appear to be able to afford CHW programmes without having to rely on external funding tend to be those with higher levels of economic development based on our proxy measure of per-capita GDP. Even in these countries, affordability may not mean provision, as doing so may require resources to be reallocated from elsewhere in the health system: the total healthcare budget must pay for hospitals, out-patient care (which would include any CHW programme), medical goods, devices and pharmaceuticals, public health, administration and research, education and training. Affordability is least likely in countries with a lower level of economic development (GDP per capita below US$1200). Thus, in the poorest countries, solely central-government-funded, full-scale comprehensive CHW programmes for the rural population are not yet a realistic solution to the human resources for health crisis. Such disparities in affordability for such a basic level of healthcare inevitably raise questions regarding equity: our work is intended to highlight such issues rather than to provide a justification for them.

Given general views that CHW programmes are inexpensive, it is important to consider why the relative cost of CHW programmes is high in relation to the funding available for healthcare. One such reason is the sheer scale of a CHW programme that provides universal healthcare access for the rural population of a country: while per inhabitant cost is low, the total cost of CHW programmes is high. There is a similar issue with primary care systems (which incorporate CHW programmes) in general. McCord *et al* suggest that such a system would cost around US$55 per capita,^13^ but of the 37 countries included in our analysis, only 10 spent this much on *any* form of healthcare in 2012.^21^ The significant challenge of meeting the global need for human resources for health (including CHWs) has been recognised by WHO, who state that ‘the scale-up required… to meet increasing demand, address existing gaps and counter expected turnover is greater than all previous estimates’.^4^

What, therefore, are the options in countries where affordability is currently low? The first option is to increase the funding available for CHW programmes, although raising additional domestic funds may not be possible in the poorest countries, which have very small tax bases. Some reallocation of funds within the health service (or across government departments) or efficiency savings could be achieved, but as noted above, this may not be easy in practice.^27 28^ Any redistribution also needs to be careful not to ‘rob Peter to pay Paul’ because different components of the health service are partly complementary to each other. Innovative methods of financing,^8^ increased publicity and awareness among donors regarding the positive impact of CHW programmes could raise additional external funds. However, a CHW programme provider needs to know that such funding will be sustained until the country has reached the level of economic development required to take over financing if potential health gains are to be realised.^18^ Changing attitudes to investments in human resources so that investment in the health workforce is seen as investment in a productive sector^4^ may also help to encourage investment.

The second option is to reduce the cost of CHW programmes. Costs can be reduced by limiting the scope of CHWs’ roles (ie, focusing on one or two key tasks/disease areas). This would enable CHWs to work at a lower coverage ratio, such as 1 to 1000 population rather than 1 to 650, but requires a comparative economic analysis of where CHWs would add most value to population health. Alternatively, models in which CHWs earn commission on the sale of medical or other goods reduce net programme costs to the provider/payer, but such models could distort CHW behaviour from what is optimal for their clients.^29^ We included CHWs as volunteers as one of our CHW salary options, and others would question the wisdom and long-term feasibility of this option.^8 14^ We support the payment of salaries but appreciate in some countries that CHWs are willing to volunteer as the alternative is no CHW programme at all (eg, Uganda^15^). Other approaches to cost-cutting, such as reducing monitoring and supervision, are unlikely to be an appropriate solution given the potential detrimental impact on the quality of the care provided.^30^ Total costs could also be reduced by not introducing CHW programmes in areas currently without any CHW provision. In the longer term, this would be challenging to justify as any existing inequality of provision, even within a country, would remain. However, in the short term, this option provides an opportunity to test out various approaches to CHW deployment before undertaking large-scale roll-out that would be difficult and expensive to alter. Of course, the optimal solution in one country may not be optimal in another, for a variety of reasons, including local norms and customs as well as access to finance.

Our work is limited by its reliance on secondary data, which is mostly from 2012. Lu *et al* 16 also highlight discrepancies between WHO and International Monetary Fund data on public spending on healthcare. Although we have used a published and robust source of CHW programme costs,^13^ this focuses on CHW programmes for the rural population. The cost of such programmes may be higher than for those in urban areas because of the time taken for CHWs to travel between households. Furthermore, the CHW programme as described is not a ‘one size fits all’ solution (as was clearly noted by McCord and colleagues^13^). In practice, there is a wide variety of CHW programme designs—in terms of tasks assigned and CHW coverage ratios—both within and between countries. More flexible costing tools that can be tailored to local contexts are available for programme providers, such as that produced by Management Sciences for Health.^31^ CHWs’ roles are also likely to evolve over time as economies grow, populations become more able to self-manage some aspects of their own health, as the burden of disease changes and perhaps as CHWs begin to be professionalised with formal training and even accreditation requirements. As in Ethiopia, new volunteer roles could emerge as the original CHW cadre starts to take on more responsibility and get paid. On the other hand, it is also plausible that CHWs as a provider of ‘first-line’ healthcare are a temporary phenomenon, bridging the gap until a full-scale professional workforce can be supported. We recognise that the labour inputs provided by CHWs (particularly in terms of hours worked but also the effort made during those hours^29 30 32^) may vary across the three salary structures included in our analysis and this would cause the non-salary costs of a CHW programme (as well as its outcomes) to vary correspondingly. For example, CHWs working longer hours are likely to require more medical supplies.

Our analysis does not incorporate the cost savings to the health system, microeconomic benefits to CHWs’ clients or macroeconomic growth that may be realised from investing in a CHW programme that has a beneficial effect on population health. Valuation of such effects would be requirement for a full, societal-level cost–benefit analysis of CHW programmes, which is beyond the scope of this paper. In addition, we did not attempt to apportion external funding for healthcare to different programmes: CHW programmes may well attract a disproportionately high share of such funding given their focus on improving access to healthcare.

With careful and ongoing consideration of how CHWs’ contributions can be maximised in relation to current and expected future social norms and disease burdens, CHWs can play a vital role in improving health outcomes. However, they cannot be effective in a vacuum—without links into the formal healthcare system—and they should not be viewed or implemented as healthcare ‘on the cheap’.^8^ Indeed, for many countries in sub-Saharan Africa, full-scale, comprehensive CHW programmes are currently not cheap at all. However, we hope that our results will initiate national-level discussions about how healthcare budgets can be increased to meet Abuja Declaration targets, best supplemented with sustainable and sensible foreign aid and subsequently allocated across services to maximise health outcomes, while working towards universal access. Such discussions need to recognise a country’s current level of economic development and consider plans for reducing reliance on foreign aid and/or future expansion of CHW programmes as the economy grows.
